# categoryCompare, an analytical tool based on feature annotations

**DOI:** 10.3389/fgene.2014.00098

**Published:** 2014-04-29

**Authors:** Robert M. Flight, Benjamin J. Harrison, Fahim Mohammad, Mary B. Bunge, Lawrence D. F. Moon, Jeffrey C. Petruska, Eric C. Rouchka

**Affiliations:** ^1^Department of Molecular and Cellular Biochemistry, University of KentuckyLexington, KY, USA; ^2^Department of Anatomical Sciences and Neurobiology, University of LouisvilleLouisville, KY, USA; ^3^Department of Neurological Surgery, Kentucky Spinal Cord Injury Research Center, University of LouisvilleLouisville, KY, USA; ^4^Department of Pathology, Beth Israel Deaconess Medical Center, Harvard Medical SchoolBoston, MA, USA; ^5^Miami Project to Cure Paralysis, Department of Neurological Surgery and Neurology, University of Miami Miller School of MedicineMiami, FL, USA; ^6^Neurorestoration Group, Wolfson Centre for Age-Related Research, King's College LondonLondon, UK; ^7^Department of Neurological Surgery, University of LouisvilleLouisville, KY, USA; ^8^Bioinformatics and Biomedical Computing Laboratory, Department of Computer Engineering and Computer Science, University of LouisvilleLouisville, KY, USA

**Keywords:** meta-analysis, comparative analysis, transcriptomics, metabolomics, proteomics

## Abstract

Assessment of high-throughput—omics data initially focuses on relative or raw levels of a particular feature, such as an expression value for a transcript, protein, or metabolite. At a second level, analyses of annotations including known or predicted functions and associations of each individual feature, attempt to distill biological context. Most currently available comparative- and meta-analyses methods are dependent on the availability of identical features across data sets, and concentrate on determining features that are differentially expressed across experiments, some of which may be considered “biomarkers.” The heterogeneity of measurement platforms and inherent variability of biological systems confounds the search for robust biomarkers indicative of a particular condition. In many instances, however, multiple data sets show involvement of common biological processes or signaling pathways, even though individual features are not commonly measured or differentially expressed between them. We developed a methodology, categoryCompare, for cross-platform and cross-sample comparison of high-throughput data at the annotation level. We assessed the utility of the approach using hypothetical data, as well as determining similarities and differences in the set of processes in two instances: (1) denervated skin vs. denervated muscle, and (2) colon from Crohn's disease vs. colon from ulcerative colitis (UC). The hypothetical data showed that in many cases comparing annotations gave superior results to comparing only at the gene level. Improved analytical results depended as well on the number of genes included in the annotation term, the amount of noise in relation to the number of genes expressing in unenriched annotation categories, and the specific method in which samples are combined. In the skin vs. muscle denervation comparison, the tissues demonstrated markedly different responses. The Crohn's vs. UC comparison showed gross similarities in inflammatory response in the two diseases, with particular processes specific to each disease.

## Introduction

With the plethora of publicly-available high-throughput molecular biology data sets including DNA microarray studies (Barrett et al., 2011; Parkinson et al., 2011), next-generation sequencing (Sayers et al., 2011), metabolomics (Fiehn et al., 2005; Scholz and Fiehn, 2007) and proteomics (Vizcaino et al., 2009), individual research groups have the potential to supplement their own experimental data with additional data sets on similar or related measurement paradigms (meta-analysis), or compare their data set with others (comparative-analysis).

Although a comparative-analysis is a type of meta-analysis, the end goals of the analyses are often different, and the summary statistics are frequently used in diverse ways. A true meta-analysis combines multiple data sets that measure the same or similar end-points to increase the accuracy of “effect size” estimates and reduce the incidence of false positives, thereby increasing the likelihood of finding robust true positives. In a comparative-analysis, the important features (e.g., differentially expressed genes) in each data set would be determined and then compared. Most meta- and comparative-analysis methods require either that the features (probes/probesets, genes, proteins, metabolites) across experiments are identical (i.e., the same or similar measurement platform is used, such as a specific Affymetrix® GeneChip™), or can be mapped directly to the same biological entity (e.g., map Affymetrix® and Agilent® probes to the same Entrez gene). In both cases, only those features commonly found across experiments will be used for subsequent analysis. Even if the same underlying biological process or pathway is impacted in multiple experiments, the above limitations may make the elucidation of common and different biological processes across data sets challenging.

Methods commonly used to summarize and infer relationships from high-throughput experiments include the set-based threshold-based (SBTB) and rank-based threshold-free (RBTF) methods. SBTB methods define a “set” of features using a “threshold.” A typical set might be differentially expressed genes with a particular *p*-value or log-fold change. The goal is then to find over-represented feature annotations (e.g., GO terms) in the set compared to all of the measured features (e.g., all genes measured). In contrast, RBTF methods do not set a threshold but rather the features are “ranked” according to an appropriate statistic, and feature annotations are tested to determine if they are significantly enriched at either extreme of the ranked list.

Commonly used feature annotations include Gene Ontology (GO) (Ashburner et al., 2000), Kyoto Encyclopedia of Genes and Genomes (KEGG) pathways (Kanehisa and Goto, 2000), chromosomal positions, transcription factor binding sites, microRNA targets, and medical subject heading (MeSH) terms. Although most feature annotation schemas annotate only one kind of feature (e.g., genes in GO), some annotate multiple feature types (e.g., genes and metabolites in KEGG). Many different tools exist to perform these types of enrichment analyses, the most widely used tools for SBTB and RBTF being GO::TermFinder (Boyle et al., 2004) and Gene Set Enrichment Analysis (GSEA) (Subramanian et al., 2005), respectively. Other tools, such as DAVID (Huang et al., 2009), provide methods to further summarize and group annotations via semantic similarity. ConceptGen (Sartor et al., 2010), in addition to considering feature annotations, also provides a comparison to determine how experimental data sets are related by comparing feature (gene) lists. This allows data sets to be defined by their list of differentially expressed genes, which are then checked against a compiled set of experimental gene lists to determine whether an association to the gene list being analyzed exists. However, in general it is not easy to compare the annotations from two or more experiments, let alone combine results from experiments that examine different types of features, such as genes and metabolites, and draw meaningful and statistically-valid conclusions.

To overcome the limitation of performing comparative analyses by considering only significant features across experiments, we sought to develop a tool which would elucidate more information about the similarities and differences between multiple high-throughput experiments by comparing the enriched feature annotations from each feature list. Here we describe a general methodology and framework for the use of enriched annotations to compare and contrast the results of high-throughput molecular biology experiments. The merits of the approach for both SBTB and RBTF types of analyses are examined using hypothetical data. The utility of the approach is demonstrated by examining the responses of skin and muscle to denervation, as well as differences between colon tissue samples in Crohn's disease and ulcerative colitis.

## Materials and methods

### categoryCompare algorithm

Given feature lists (such as genes) (F_1_, F_2_,…, F_n_) resulting from multiple high-throughput experiments and their annotations (such as GO terms), *p*-values for all the annotations in each feature list are calculated. Significant annotations for each list are filtered using a pre-specified *p*-value and a minimum number of annotated features, resulting in a series of annotation lists (A_1_, A_2_, …, A_n_), each corresponding to a feature list. From the set of enriched annotation lists, a “list membership” can be determined using any or all of the 2^n^-1 non-empty subsets of annotation lists. For example, if four feature lists (F_1_, F_2_, F_3_, and F_4_) are supplied, four corresponding enriched annotation lists are determined (A_1_, A_2_, A_3_, and A_4_). In this example, there are 2^4^−1 = 15 possible non-empty subsets of annotation lists for determining “list-membership” including: {A_1_}, {A_2_}, {A_3_}, {A_4_}, {A_1_,A_2_}, {A_1_,A_3_}, {A_1_,A_4_}, {A_2_,A_3_}, {A_2_,A_4_}, {A_3_,A_4_}, {A_1_,A_2_,A_3_}, {A_1_,A_2_,A_4_}, {A_1_,A_3_,A_4_}, {A_2_,A_3_,A_4_}, and {A_1_,A_2_,A_3_,A_4_}. The final lists of enriched annotations, along with the statistics from each feature-list are supplied in a table for examination.

### Annotation graphs

As a means of working with both the large number of possibly redundant annotations and highly similar annotations that may have different list-memberships, the annotations are also reported as a graph relation similar to that implemented in Enrichment Map (Merico et al., 2010). In this representation, each node in the graph is an annotation, and edges between annotations are weighted by the degree of feature overlap between annotations. Feature overlap is calculated using either the Jaccard (general annotations), overlap (gene ontology), or combined (user defined) coefficient (Merico et al., 2010). Information about the annotations (ID, description, list membership, *p*-values, etc.) is stored as meta-data for each node. In contrast to a long table of annotations, the graph structure facilitates determination and visualization of clusters of highly related and possibly redundant annotations with various list-memberships. The annotation graphs can then be combined with a graph visualization framework (such as Cytoscape), nodes can be colored by their “list-membership,” or represented by a pie-chart, wherein each slice represents a separate sample list, with significance in the list denoted by assigned color, and non-significance by de-saturated color.

Alternatively, for GO term annotations, the annotation list-membership may also be determined based on the induced ancestor graph generated from the significantly enriched GO terms from each feature-list. This allows exploration of the results in the GO-directed acyclic graph, while maintaining information about the location of the terms in the GO hierarchy.

### Implementation

categoryCompare has been implemented using the R statistical language (Ihaka and Gentleman, 1996) as a Bioconductor (Gentleman et al., 2004) package (http://bioconductor.org/packages/release/bioc/html/categoryCompare.html), allowing users to easily integrate it into high-throughput data analysis workflows, and facilitating the use of the many different gene annotation packages available in Bioconductor. The package supports the direct calculation of enriched GO and KEGG annotations on gene lists. Interactive visualization of the annotation graphs is facilitated using the RCytoscape (Shannon, 2011) Bioconductor package to display the graph in Cytoscape (Shannon et al., 2003).

## Muscle vs. skin denervation

### Skin denervation

All procedures were approved by the Institutional Animal Care and Use Committees of the University of Louisville and SUNY Stony Brook and were in accord with the guidelines set forth by the NIH (Institute of Laboratory Animal Resources et al., 1996) and the American Veterinary Medicine Association (Packer et al., 2001). Adult female Sprague-Dawley rats (160–200 g; Taconic, Hudson, NY) were used. Control animals (naïve) were not treated. Those animals that underwent skin denervation surgery were anesthetized with 60 mg/kg pentobarbital administered intraperitoneally. Body temperature was monitored with a rectal thermistor and was maintained at 35–37 degrees C with a heated water pad. EKG leads were also placed to monitor heart rate. Incision of the dorsal skin from T4 to L5 on the contralateral (right) side was performed to allow reflection of the skin over to the ipsilateral (left) side to expose dorsal and lateral cutaneous nerves (D/LCNs). This surgical approach prevented damaging the left-side skin. Two areas of denervated skin were produced by transection of the T9/10 and T12/13 DCNs.

All proximal nerve stumps were then sealed off with ligatures close to the body wall to prevent their regeneration. The T11 D/LCNs were left intact producing an island of intact innervation. These intact axons provided collateral sprouts which projected from their home dermatome into the surrounding denervated skin (e.g., Diamond et al., 1992). Incisions were then sutured and stapled and the denervated areas mapped acutely, using permanent marker on the skin to define the border between innervated and denervated areas, by determining the ability of pinch applied to the skin to drive the cutaneous trunci muscle (CTM) reflex as previously described (Theriault and Diamond, 1988; Diamond et al., 1992; Petruska et al., 2014). Maps were maintained with regular re-application of border marks.

After 7 or 14 days (corresponding to the initiation and maintenance phases, respectively, of axonal collateral sprouting), animals were euthanized with an overdose of pentobarbital and the rostral-most skin samples corresponding to the acutely mapped zones (i.e., those regions denervated by transection of the T9 and T10 cutaneous nerves) were removed and then trimmed to approximately 100 mg before RNA extraction using 1.5 ml Trizol reagent (Invitrogen, Carlsbad, CA) by homogenizing using a Polytron-style rotor-stator and then centrifuging at 12,000 g for 10 min at 4°C. RNA was then extracted from the supernatant as per the manufacturer's protocol. RNA pellets were dissolved in 50 ul nuclease-free water before purity assessment using UV spectrometry (A260 nm/A280 nm > 1.9, A260/A230 > 2.0).

RNA quality was assessed by formamide-gel electrophoresis. Biotin-labeled fragmented cRNA probes were then synthesized and hybridized to Affymetrix® rat gene expression Rat Genome 230 2.0 GeneChip™ microarrays as per manufacturers protocols. This data is available from GEO as series GSE54356.

### Muscle denervation

The mouse muscle denervation normalized microarray data was obtained directly from the NCBI GEO databank (GEO GSE4411). See Wang et al. (2005) for a description of the generation of tissue samples.

### Crohn's and ulcerative colitis

The Crohn's and ulcerative colitis (CUC) data was obtained directly from the NCBI GEO databank (GEO GSE36807). See Montero-Melendez et al. (2013) for a description of the samples.

### Microarray data processing

The skin denervation data set consisted of arrays from naïve (innervated) rats (*n* = 6) and arrays from rats 7 days (*n* = 5) and 14 days (*n* = 5) after denervation. These times correspond roughly to the initiation and maintenance phases of the axonal collateral sprouting (CS) process, respectively (e.g., Diamond et al., 1992), and are times at which denervation-induced epidermal thinning is evident (Nurse et al., 1984; Hsieh et al., 1997). The muscle denervation data set had three samples from control (innervated) mice and three from mice 3 days following denervation of the tibialis anterior muscle by transection of the sciatic nerve (GEO GSE4411, Wang et al., 2005). The skin microarray data were normalized using robust multi-array averaging (RMA). The muscle microarray data was obtained from GEO already normalized and log-transformed. For both, control probesets were removed, and log fold-changes and *p*-values of differential expression at each day (7 and 14 for skin, 3 for muscle) compared to naïve calculated using LIMMA (Smyth, 2005). Probesets were mapped to Entrez IDs using version 2.10.1 of the “org.Rn.eg.db” and “org.Mm.eg.db” packages from Bioconductor (v 2.13). See the “Skin vs. Muscle” vignette in the ccPaper package (https://github.com/rmflight/ccPaper) for further details.

The Crohn's and ulcerative colitis (UC) dataset consists of intestinal biopsy samples from healthy (*n* = 7), Crohn's (*n* = 13), and ulcerative colitis (*n* = 15) individuals (Montero-Melendez et al., 2013). The data were obtained already normalized and log-transformed. Log fold-changes and *p*-values of differential expression of both Crohn's and UC compared to normal samples were calculated using LIMMA. Probesets were mapped to human Entrez IDs using version 2.10.1 of the “hgu133plus2.db” package from Bioconductor. See the “UC vs. Crohn's” vignette in the ccPaper package (https://github.com/rmflight/ccPaper) for further details.

For those Entrez genes with multiple probesets, the probeset expression levels were collapsed to a single value by taking the median intensity of all probesets for a gene. Any probesets that did not map to genes or mapped to multiple genes were removed from further consideration.

### Annotation enrichment

Enriched annotations were calculated using LIMMA's romer method with 10,000 rotations and GO biological process (GO::BP) defining the gene sets. A *p*-value cutoff of 0.01 was used to determine significant GO::BP terms in both the “Up” and “Down” results from romer for each experiment. Major GO term groups were determined by visualizing and inspecting the GO terms and their connections in Cytoscape. Inspection of each group by the authors determines an overall naming for the group of terms. For visualization in Cytoscape, edges with an overlap score less than 0.8 were removed from the annotation graph. The reported “name” for each group of enriched annotations was reported by the authors' in an attempt to summarize commonalities among the GO terms in a specific group. The full list of GO terms for each gene list is available in the supplemental materials, as well as the GO terms in each group.

### Hypothetical data sets

A full description of the hypothetical data generation and analysis is available in the vignette “hypothetical example” of the ccPaper R package (https://github.com/rmflight/ccPaper). A summary of both the set-based threshold-based and rank-based threshold-free hypothetical datasets follows.

### Set-based threshold-based (SBTB)

All gene-ontology terms within the biological process (GO::BP) category for *Homo sapiens* and the annotated genes were considered. One-hundred GO::BP terms from the resulting set were selected to define the biological processes present in hypothetical samples; 50 with 10–100 genes annotated, 30 with 250–500 genes annotated, and 20 with 500–1500 genes annotated (these values were chosen according to the distribution of annotated genes to GO terms shown in Figure 2A). Based on this set of 100 GO::BP terms, hypothetical samples of 1000 human genes annotated to the 100 GO terms was generated using a distribution-based sampling routine. The sampling was performed as follows: for a given GO::BP term, the number of genes sampled from that term was randomly determined based on a random number from an exponentially decaying function multiplied by the total number of genes annotated to that term, without considering those genes already sampled. The chosen genes are then removed from consideration for all GO::BP terms in which they are annotated. Each GO::BP term is considered in turn until the limit of 1000 genes is reached. For all further experiments, two independently generated samples of 1000 genes based on identical sets of GO terms were used to represent significantly differentially expressed genes from two independently performed experiments on the same biological system.

Hypergeometric *p*-values were calculated for the 100 GO::BP terms for each sample independently as well as a combined sample consisting of the intersection of the sample lists. For each GO::BP term, the *p*-value was transformed by taking −1^*^log of the *p*-value, and the difference between the minimum transformed *p*-value from each sample and the combined list was reported.

The dependence of the *p*-value and difference on gene samples was estimated by repeating the calculation using 100 different gene samples. Dependence on the GO::BP term set used was estimated using 100 different GO::BP term samples.

The effect of adding noise genes (genes not annotated to the 100 GO::BP terms selected) was assessed by adding variable numbers of noise genes (10–1000, in increments of 10), and in each case varying the fraction of noise genes that were shared between two samples with the same number of noise genes (0 to 1, in increments of 0.01).

To verify that the distribution of genes to GO::BP terms from the hypothetical SBTB significant gene list was reasonable, two data sets were considered: the acute lymphocytic leukemia (ALL) data set (Li, 2009) available as a Bioconductor package, and a paired case-control lung cancer dataset from GEO (LUNG – GSE18842) (Sanchez-Palencia et al., 2011). In both cases, LIMMA was used to calculate significantly differentially expressed genes. For ALL, comparisons were made between B and T cell ALL. For LUNG, a cancer to control sample comparison was made. The resulting distributions for both of these sets show a reasonable match to the proposed hypothetical distribution (results not shown).

### Rank-based threshold-free (RBTF)

The directed acyclic graph relationship of GO:BP terms makes simulating RBTF samples using GO::BP as the gene sets challenging. Therefore, a hypothetical set of independent terms was created. Independent terms were identified by the number of genes annotated to the term, defined using a uniform distribution over a specified range, using three groups of ranges with similar limits as in the SBTB case, namely 20–250 (50 terms, low), 250–500 (30 terms, med) and 500–1000 (20 terms, hi). Each term used the same uniform distribution of 10,000 values prior to assignment of gene indices. *P*-values were on the interval [0, 1], whereas t-statistics were on the interval [−6, 6]. Different proportions of genes in the top 50% of entries were defined using a uniform distribution on the range of [0.3, 1]. For each term, a random set of indices in the rank list were determined, and then for each sample a random set (defined by the proportion in the top 50% for that term) of those indices were assigned values from the upper 50% of ranked statistics, and the rest from the bottom 50%. This results in an independent set of statistics for each sample for each term. LIMMA's geneSetTest was used to calculate probabilities of the chance of the gene set being at the “lower” extreme of the ranked list by chance by comparing the average value of statistic of the set against 10,000 random samples the same size from the same ranked list of statistics. Prior to use with geneSetTest, a linear transformation was performed so that *p*-values were in the range of [−1, 1] to enable testing in geneSetTest of annotations being at the “end” of the ranked list. A combined sample was generated by combining the gene-level statistics by one of: averaging t-statistics; Fisher's method for *p*-values; the maximum *p*-value between samples.

Differences between sample-based and combined annotation term *p*-values were calculated as the difference of the minimum of the −1^*^log(P) for sample and −1^*^log(P) for combined.

## Results

**Figure 1:**
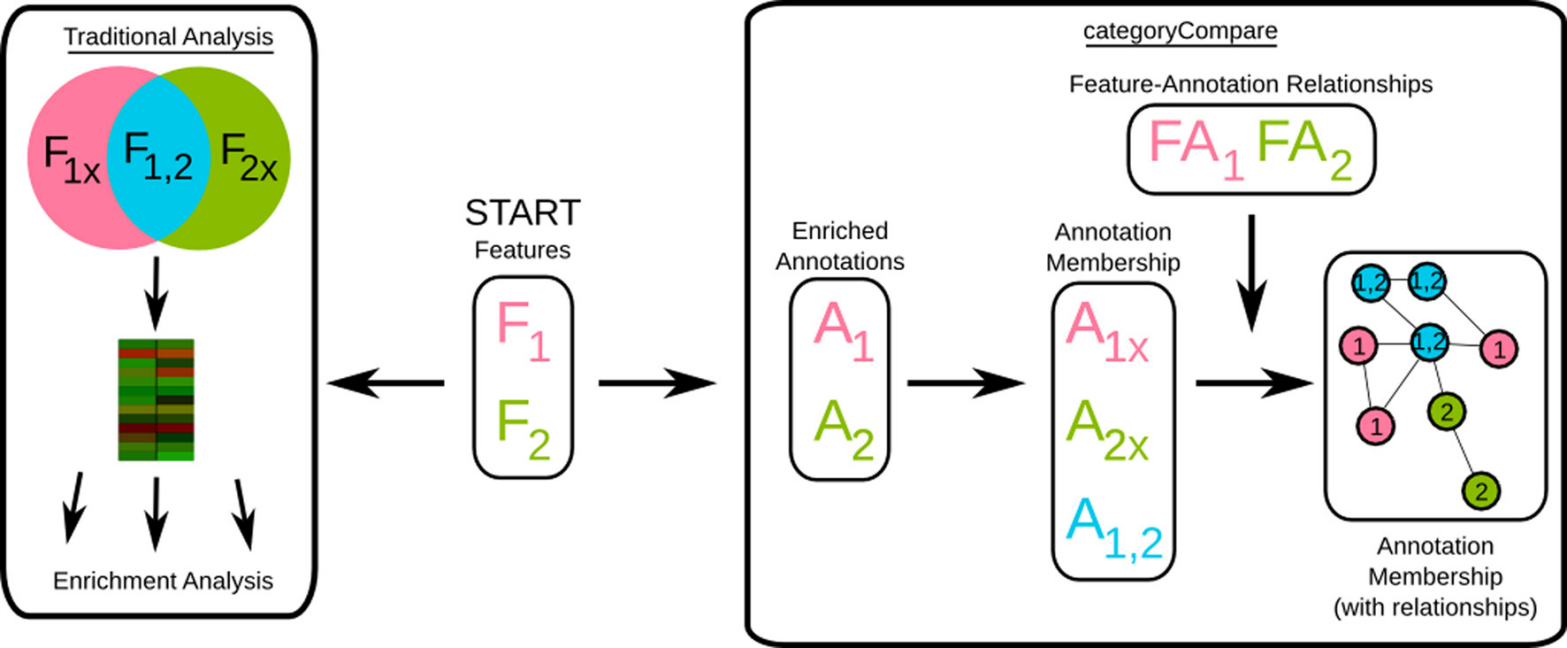
**Flow diagram of comparative-analysis approaches**. F, feature list; A, annotation list; FA, feature to annotation relationship data; 1,2, data set origination. “Start” denotes initial data availability, in this case from two different data sets, F1, and F2. The “Traditional Analysis” considers feature intersection and set-differences, possibly combined with direct comparison of the features (shown as a heatmap); whereas categoryCompare uses the enriched annotations of the feature list from each data set derived independently, combined with the feature-annotation relationships. See Results and Methods for more details.

Traditional methods for comparing high-throughput data sets depend on having shared features between the data sets for comparison. A simple first level analysis is to compare the expression levels of shared features (shown as a heatmap of genes in two data sets in Figure 1). This may be followed by some form of enrichment analysis on the overlapping feature sets to determine biological themes among the shared features (and possibly on the unique feature lists as well, see “Traditional Analysis” in Figure 1). However, there are many different scenarios that complicate this analysis, including cases when different features are measured (e.g., two studies using different microarray platforms), or comparing studies that examine different types of features with the same annotations (transcriptomics vs. metabolomics for example). In addition, experimental and biological variability may result in differences in final measurements among experiments, even though the same biological process or pathway may be involved. In these situations, it may be more effective to compare and contrast feature sets based on their enriched annotations, rather than the features directly.

Figure 1 illustrates this contrast. As a simple example, consider two feature lists from two different data sets (“Start,” middle box of Figure 1). In the “Traditional Analysis,” the intersection and set-difference of the features are determined, and feature level comparisons made. Biological summaries of the intersection and set differences are provided by annotation enrichment of each of the resulting lists. In categoryCompare, enriched annotations are calculated for each independent feature list (A), and “list-membership” assigned based on which feature list produced which annotation (in this case, x denotes an annotation or feature was found exclusively from a particular list).

### Hypothetical data

In an effort to determine how results using categoryCompare differ from the “traditional analysis,” hypothetical datasets for both SBTB and RBTF analysis situations were generated (see Methods for the details of the generation of data sets and calculation of enriched annotations or terms).

**Figure 2:**
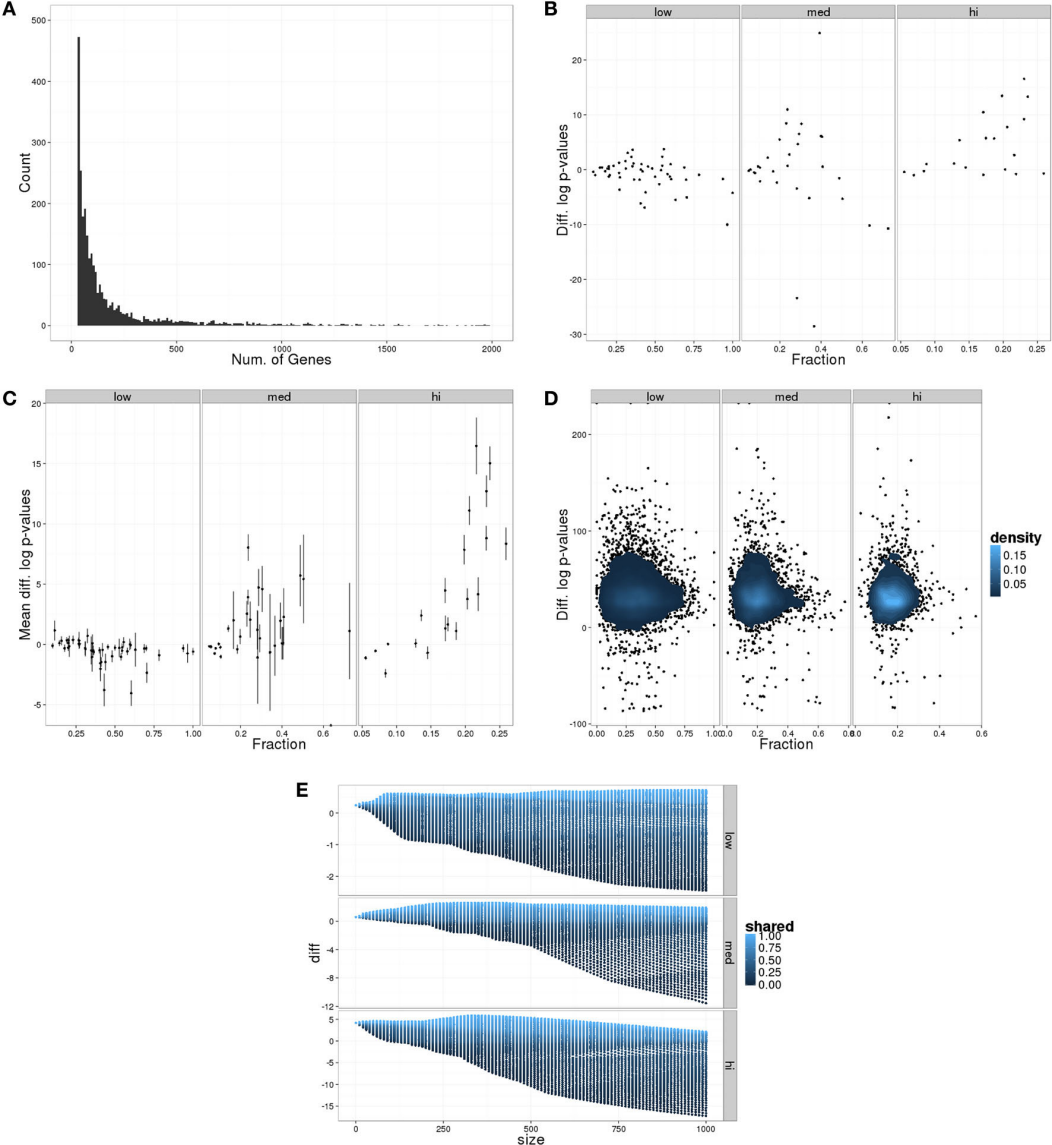
**Results of SBTB hypothetical data analysis**. **(A)** Distribution of number of genes annotated to GO::BP terms in human. **(B)** Difference in transformed *p*-values comparing maximum *p*-value from sample calculations and *p*-value from combined sample as a function of the fraction of genes in the sample annotated to the GO term. Plot is divided by the size classification of the GO term. **(C)** Average and standard deviations of performing the same calculations as in **(B)** using 100 independent sample generations. **(D)** Contour plot after using 100 different GO::BP sets to perform the same calculations as in **(B)**. **(E)** Median *p*-value differences (taking the median using size as the grouping) as a function of the number noise genes added, colored by the fraction of noise genes shared between two samples.

For the SBTB analysis, two hypothetical samples of 1000 genes were sampled from 100 GO terms to represent differentially expressed genes (the set) from two different experiments where the same fundamental biological processes were responsible. Figure 2B shows how the transformed *p*-value calculated from the combination of the samples (differentially expressed gene list is the intersection of the two samples) compares to the minimum transformed *p*-value for that GO term from the two samples. In many cases, the sample wise *p*-values are much better than those from the combined sample; however, this appears to be dependent both on how many genes are annotated to the term and the proportion of genes annotated to the term in the differentially expressed set.

The reproducibility of these results are shown in Figures 2C,D, where the calculations were repeated using the same GO terms but different gene samples 100 times (Figure 2C), and using 100 different samplings of GO terms (Figure 2D). For gene samples, the results are relatively stable. In contrast the GO sampling shows much wider variability, but the trend remains stable across the different GO::BP samples.

The effect of noise genes (genes not annotated to any of the GO terms under consideration) was assessed by varying both the total number of noise genes (10–1000 incremented by 10) and the fraction of noise genes that are shared between the two samples (0–1 incremented by 0.01). Figure 2E shows the median difference between the minimum transformed sample *p*-value and combined *p*-value as a function of the number of noise genes added, colored by the fraction of shared noise genes. As can be observed, in general the sample wise *p*-values do better when there are more noise genes shared between the samples; however, the difference can be large depending on the number of genes annotated to the GO term (denoted by the size class of the terms as either low, med, or hi).

**Figure 3:**
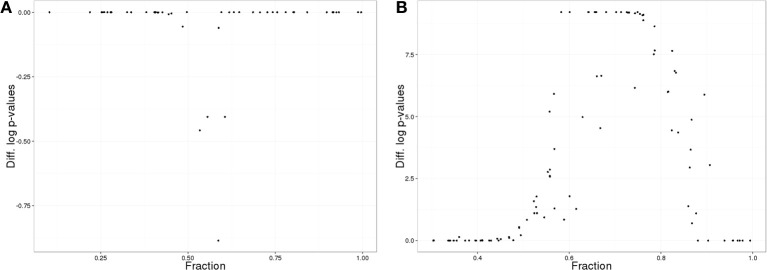
**Results of RBTF hypothetical data analysis**. **(A)** Difference of transformed *p*-values comparing the maximum term *p*-value from sample calculations and *p*-value from a combined sample where samples were combined using averages of t-statistics, as a function of the proportion of genes in the top 50% of entries by rank. **(B)** As in **(A)**, but the maximum *p*-value was used to combine samples gene-wise.

Given the challenges involved in generating appropriate ranks for the RBTF method for annotations with a directed acyclic graph structure such as GO, independent theoretical terms and ranked statistics for two hypothetical samples in each term were generated (see Methods). Generating combined samples was achieved by averaging t-statistics, using Fisher's method to combine *p*-values, and taking the maximum *p*-value across samples. Figure 3A shows the difference in term enrichment *p*-values between the minimum transformed *p*-value from the samples and the transformed *p*-value from the combined sample where the sample was combined using an average of t-statistics. As the proportion of genes in the top 50% of ranks varies, the combined sample gives at least as good or better *p*-values, until a threshold proportion is reached. Combining samples using Fisher's method generated similar results (results not shown). Combining samples using the maximum *p*-value across samples demonstrated completely opposite behavior, with the combined sample yielding worse *p*-values (Figure 3B).

### Skin vs. muscle denervation

The categoryCompare approach was used to compare gene expression following denervation of skin and muscle. The denervated skin data set consisted of DNA microarray measurements from rats, comparing the transcript expression in the naïve skin, and skin at 7 and 14 days following denervation. The muscle data set consisted of DNA microarray samples of control muscle and muscle 3 days post-denervation from mice (Wang et al., 2005).

For each of the comparisons (Skin.T7, Skin.T14, Muscle) those annotations with a reported *p*-value less than 0.01 for either changes up or down were used for subsequent analysis, resulting in six different annotation lists. The number of significant annotations in each list is reported in Table 1.

**Table 1:** **Number of significantly enriched annotations in each list following denervation**.

**Skin.T7.Down**	**Skin.T7.Up**	**Skin.T14.Down**	**Skin.T14.Up**	**Muscle.Down**	**Muscle.Up**
263	343	247	259	151	130

Analysis of the 79 significantly enriched annotation groups shows that the majority of these (59) respond to a single condition, including 10 in Skin.T7.Down (such as negative regulation of astrocyte differentiation, and collateral sprouting); fifteen in Skin.T7.Up; eight in Skin.T14.Down; nineteen in Skin.T14.Up (including synapse assembly and nervous system development, axon extension, and glia guided migration); five in Muscle.Down (including response to VEGF); and three in Muscle.Up. There are two annotations down-regulated in all three data sets (ATP biosynthesis and muscle adaptation and contraction); one annotation down-regulated in skin and up-regulated in muscle (mitotic cell cycle checkpoint); one down-regulated in Skin.T7 and upregulated in Skin.T14 and Muscle (pH and lysosome regulation); four down-regulated at Skin.T7 which are up-regulated at Skin.T14 (neurotransmitter secretion and transport, endocrine regulation of blood pressure, fluid transport, and epithelial tube branching); one down-regulated in Skin.T7 and Muscle (response to glucose synthesis); one up-regulated in Skin.T7 and down-regulated in Skin.T14 (microtubule organization); two up-regulated in both skin datasets (blood vessel endothelial cell differentiation and negative regulation of protein export); and three down-regulated in Skin.T14 and Muscle (cellular respiration, acetyl CoA biosynthesis and response to muscle activity). A complete list of the membership for each of the significantly enriched annotations is provided in Table 2.

**Table 2:** **Significant term annotation groups and the gene-list they appeared significant in for the Skin vs. Muscle comparison**.

**Description**	**Skin.T7.Down**	**Skin.T7.Up**	**Skin.T14.Down**	**Skin.T14.Up**	**Muscle.Down**	**Muscle.Up**
ATP biosynthesis	X		X		X	
Muscle contraction and development	X		X		X	
Mitotic cell cycle checkpoint	X		X			X
pH and lysosome regulation	X			X		X
Neurotransmitter secretion and transport	X			X		
Epithelial tube branching	X			X		
Endocrine regulation of blood pressure	X			X		
Fluid transport	X			X		
Tube development	X					X
Response to glucose	X				X	
Steriod biosynthesis	X					
Lung cell differentiation	X					
Neg regulation of astrocyte diff.	X					
Fear response	X					
Dopamine transport	X					
Collateral sprouting	X					
Response to osmotic stress	X					
Positive regulation of epidermal growth factor signaling	X					
Response to leptin	X					
Catecholamin biosynthesis	X					
Microtubule organization		X	X			
Blood vessel endothelial cell differentiation		X		X		
Negative regulation of protein transport		X		X		
Negative regulation of response to granulocyte/myeloid cell diff.		X				X
Type II hypersensitivity		X				
Entry into host and movement		X				
Response to virus		X				
Mitotic spindle assembly		X				
N acetylglucosamine metabolism		X				
Proteoglycan biosynthesis		X				
Negative regulation of cell junction assembly		X				
Endocardial cell differentiation		X				
Sequestering of actin monomers		X				
Beta amyloid formation		X				
Response to platelet derived growth factor stimulus		X				
Lipopolysaccharide biosynthesis		X				
Aminoglycan metabolism		X				
Epithelial to mesenchymal transition, endocardial cushion formation		X				
Pulmonary valve dev. and morphogenesis		X				
Cellular respiration			X		X	
acetyl CoA biosynthesis			X		X	
Response to muscle activity			X		X	
Protein localization in mitochondrion			X			
Cation channel activity			X			
Interferon gamma response			X			
Calcineurin NFAT signaling cascade			X			
Histone demethylation			X			
Negative regulation of Ras signal transduction			X			
Positive regulation of metalloenzyme activity			X			
Glucocorticoid receptor signaling pathway			X			
Atrioventricular valve dev. and morphogenesis				X	X	
Vitamin metabolism				X		X
Digestion				X		
Synapse assembly and nervous system development				X		
Folic acid compound metabolism				X		
Retinoic acid biosynthesis				X		
Urea cycle				X		
Response to prostaglandin				X		
Keratinocyte migration				X		
Amino acid transport				X		
Mesenchymal cell diff in kidney/Renal system dev.				X		
Axon extension				X		
Lung lobe dev. and morphogenesis				X		
Embryonic digestive tract dev. and morphogenesis				X		
Phosphate ion transport				X		
Fluid secretion				X		
Interleukin 13 production				X		
Glia guided migration				X		
Central nervous system maturation				X		
Bile acid transport				X		
Positive regulation of muscle cell apoptosis				X		
Purine metabolism					X	
Regulation of phospholipase C					X	
Response to VEGF					X	
Membrane repolarization					X	
Carbohydrate catabolism					X	
rRNA processing						X
Response to indole 3 methanol						X
DNA break repair						X

### Crohns vs. UC

categoryCompare was employed to compare the gene expression from Crohn's disease and ulcerative colitis (UC) intestinal samples when each was compared to normal samples. GO::BP annotation enrichment was used. Table 3 lists the number of significant GO::BP terms for each comparison using a *p*-value cutoff of 0.01.

**Table 3:** **Number of significant annotations for each comparison for Crohn's compared to UC using romer and *p*-value cutoff of 0.01**.

**UC.Up**	**UC.Down**	**CROHNS.Up**	**CROHNS.Down**
434	169	264	110

As listed in Table 3, UC up-regulated compared to normal (UC.Up) had by far the largest number of significant GO::BP terms, and this is reflected in the overall results as there are large groups of highly related GO terms listed as being significant solely or primarily in UC.Up. These include “response to lipopolysaccharide and bacterial,” “regulation of inflammatory response,” “regulation of cell cycle and DNA damage response,” “regulation of ubiquitination and ligase activity,” and “vesicle targeting.” The CROHNS.Up group had much fewer significant GO::BP terms overall, and few that were found only in CROHNS.Up. These included “NAD biosynthesis,” “hormone metabolism,” “response to growth hormone,” “melanin metabolism,” and “protein dephosphorylation.” Common to UC.Up and CROHNS.Up are “amine metabolism,” “extrinsic signal transduction,” “regulation of nitric-oxide synthase,” “fatty-acyl-CoA biosynthesis,” “chemokine and cytokine production,” and “hydrogen peroxide metabolism.” Specific to UC.Down are “glandular cell differentiation,” “membrane biogenesis and assembly,” and “activin receptor signaling.” Common to UC.Down and CROHNS.Down include “oligodendrocyte differentiation” and “cellular pattern specification.” All of the GO::BP groups are listed in Table 4 by list-membership, and the individual GO terms in each group are provided in the supplemental materials.

**Table 4:** **Significant term annotation groups and the gene-list they appeared significant in for the CROHNS vs. UC comparison**.

**Description**	**UC.Down**	**UC.Up**	**CROHNS.Down**	**CROHNS.Up**
Hydrogen peroxide metabolism		X		X
Nucleotide and nucleoside metabolism		X		X
Amine metabolism		X		X
Extrinsic signal transduction		X		X
Regulation of nitric-oxide synthase		X		X
Fatty-acyl-CoA biosynthesis		X		X
Chemokine and cytokine production		X		X
ER unfolded protein response		X		X
Antigen processing and presentation		X		X
Response to lipopolysaccharide and bacterial		X		
Regulation of inflammatory response		X		
Regulation of cell cycle and DNA damage response		X		
Regulation of ubiquitination and ligase activity		X		
nik/nk-kappab cascade		X		
Regulation of ras/rac/rho gtpase activity		X		
COPII vesicle coating and targeting		X		
Negative regulation of peptidase activity		X		
Response to type 1 interferon		X		
Protein N-linked glycosylation		X		
Glandular cell differentiation	X			
Membrane biogenesis and assembly	X			
Activin receptor signaling	X			
Oligodendrocyte differentiation	X		X	
Cellular pattern specification	X		X	
NAD biosynthesis				X
Hormone metabolism				X
Response to growth hormone				X
Melanin metabolism				X
Protein dephosphorylation				X

### Annotation graphs

**Figure 4:**
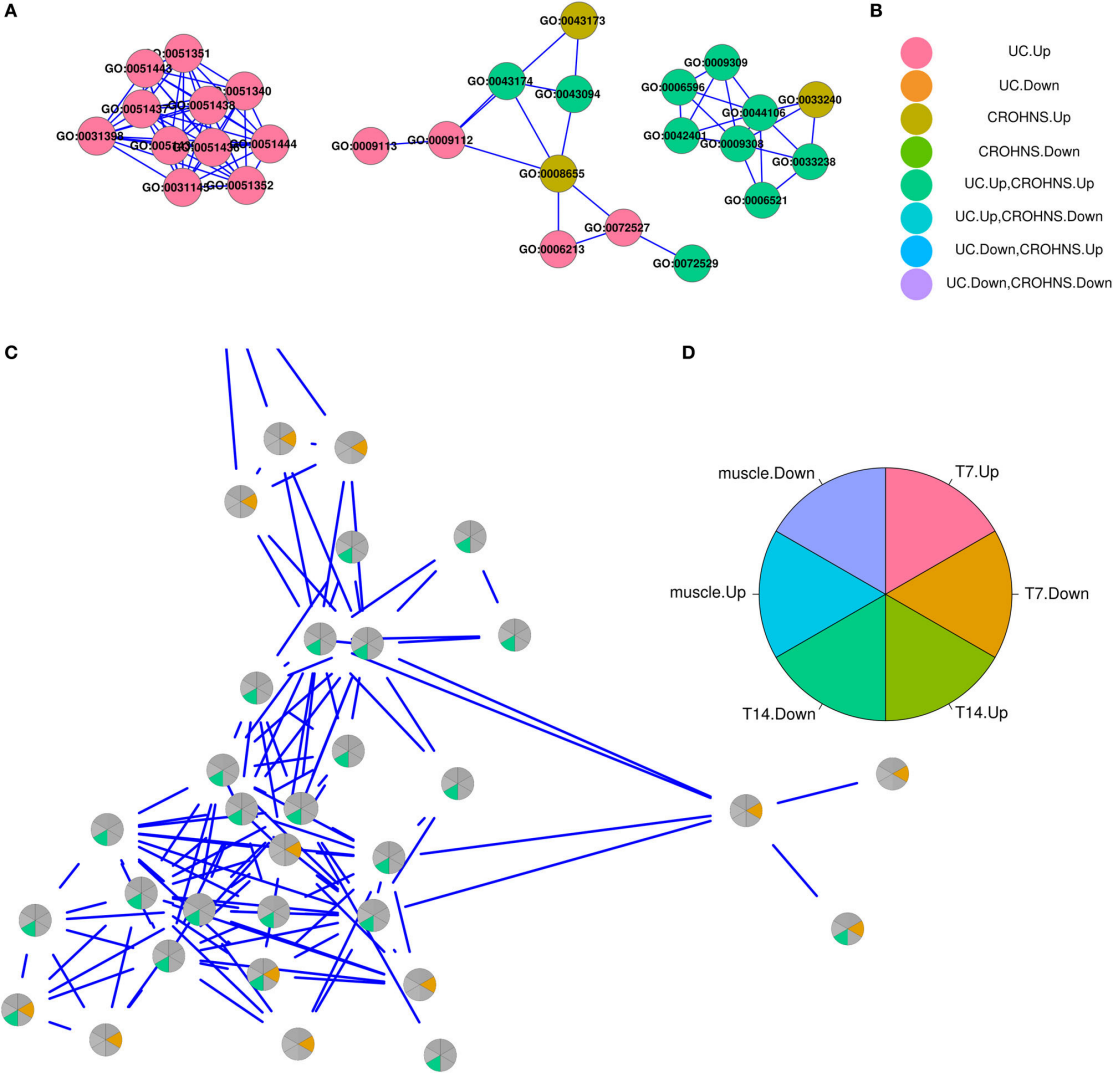
**Examples of annotations groups and associated legends**. **(A)** From left to right, visualization of annotation groups “regulation of ubiquitination and ligase activity,” “nucleoside and nucleotide metabolism,” and “amine metabolism,” with associated legend in **(B)**. **(C)** Pie chart visualization of annotation group “muscle fiber development,” with legend in **(D)**.

For both the CROHNS vs. UC and Skin vs. Muscle datasets, the enriched annotations are reported as “themes” of groups of highly similar annotations, where similarity is defined by the shared number of genes between annotations (see Methods). Figure 4 shows small examples of the visualization used to interactively determine the themes of highly related groups of annotations (only edges with a similarity ≥ 0.8 are shown). Figure 4A shows three different annotation groups from the CROHNS vs. UC analysis (from left to right): “regulation of ubiquitination and ligase activity,” “nucleoside and nucleotide metabolism,” and “amine metabolism” with the associated legend in Figure 4B. Figure 4C shows the annotation group from SKIN vs. MUSCLE “muscle fiber development,” with the associated legend in Figure 4D. Both instances demonstrate that not all of the annotations in a group necessarily have an identical “list-membership,” but rather the total “list-memberships” of the annotations are considered together to assign the “lists” that annotation group is from. For example, the middle group in Figure 4A, “nucleoside and nucleotide metabolism” has only two GO terms that were both from UC.Up and CROHNS.Up, but all the other highly related GO terms are from UC.Up or CROHNS.Up. Similarly, in the Skin vs. Muscle example, many terms are found in only one or the other of Skin.T7.Down or Skin.T14.Down, but they are all highly related to one another by the genes annotated to each term.

Although not part of the categoryCompare algorithm *per se*, returning the annotations as a graph where each node is an annotation and edges are weighted by their similarity (see Methods) enhances the biological interpretation by providing the ability to determine sets of highly related annotations that is not easily accomplished in list form. For example, of the nine annotations in the middle group of Figure 4A (“nucleoside and nucleotide metabolism”), only three have a “list-membership” of UC.Up, CROHNS.Up. If the full list of significant annotations (288) are ranked by: UC.Up *p*-value; CROHNS.Up *p*-value; and then by their list-memberships; they are scattered throughout the table, being located in rows 40, 123, 136, 149, 155, 167, 191, 206, and 215. Deriving the theme of “nucleoside and nucleotide metabolism” from the items in the list would be more difficult. It should be noted that all of the tabular data is available in Cytoscape when the user selects a node, and multiple nodes may be selected simultaneously, thus providing all the tabular output for a particular group of nodes.

## Discussion

### Crohn's vs. UC data

While the original CROHN'S vs. UC study focused on determining predictor genes as clinical biomarkers for classifying Inflammatory Bowel Disease, we employed categoryCompare to determine the similarities and differences between these two conditions at a higher annotation-based level, which is highlighted in the visualization of the GO results (Figure 4). The results suggest that both conditions are enriched for processes involved with immune response (antigen processing and presentation) as well as chronic inflammation (chemokine and cytokine production). Additionally, both datasets show an upregulated enrichment of amine, nucleotide, and nucleoside metabolism which has previously been associated with the vascular surface of inflammatory diseases (Eltzschig et al., 2004).

Differences in the enriched annotations indicate that UC has a significant up-regulated enrichment in cell-derived mediators of inflammation, including nik/nk-kappab cascade, regulation of inflammatory response, and response to type I interferon. The significant increase in the regulation of ubiquitination and ligase activity specific to UC is consistent with GWAS studies implicating loci coding for proteins with domains associated with protein ubiquitination (McGovern et al., 2010). The results from categoryCompare agree with this association, since these loci were not found to be associated with Crohn's Disease patients.

The significant increase in hormone metabolism in CROHNS may point to the difference in bone mineral density (Ardizzone et al., 2000) while the increased response in growth hormone in CROHNS may be directly related to growth hormone therapies specific to the treatment of Crohn's Disease (Slonim et al., 2000; Heyman et al., 2008).

### Skin vs. muscle denervation data

Our interests lie with the role of denervated tissue in recruiting the non-injured axons innervating adjacent tissue to send new branches through the tissue into the denervated territory (often called collateral sprouting or collateral reinnervation).

Overall the annotation groups which emerged for denervated skin and denervated muscle are largely different. This is likely due in part to the intrinsic difference in tissues, one being a barrier tissue with rapid cellular turnover that is highly sensitive to innervation status (Hsieh et al., 1997). Similarities reside in the emergence of annotations suggesting reduced energy production in both tissues as well as a concomitant increase in stress-response and stimulus-response processes in both denervated tissues.

### Hypothetical data

The hypothetical SBTB and RBTF results echo previous results from Shen and Tseng (2010). Their results showed that depending on the degree of overlap and how large the gene signal was, meta-analysis at the feature level or annotation level have different advantages. For SBTB type analyses, whether feature level combination or annotation level combination is better depends on the overall number of genes annotated to the term, as well as what fraction of genes are present in the individual samples.

For small numbers of genes annotated to a term, the likelihood of getting the same genes in both samples is likely random, thus explaining the seemingly random distribution of change in *p*-value for the small set of GO terms in the single sample, and for multiple sets of gene samples. However, when viewed over 100 sets of GO samples, the overall trend is that categoryCompare gives better *p*-values over using the sample intersection. However, as the amount of noise increases and the fraction of shared noise decreases, the intersection of genes between the two experiments becomes much more specific, leading to the intersection of lists giving much better *p*-values overall.

In contrast, with an RBTF type analyses such as GSEA, the degree of improvement using categoryCompare is dependent on how the samples are combined, in addition to the fraction of genes that are highly ranked in the samples. The dependence on the fraction is not surprising, as the odds that the set will be highly ranked as a whole becomes more likely as the overall fraction increases, with no difference at higher fractions. In addition, how the samples are combined is important in that the most conservative method, taking the maximum *p*-value (the method originally used by Shen and Tseng), should result in the lowest fraction of genes being highly ranked in the combined sample.

Both of these results suggest that categoryCompare has advantages in particular situations, but is still limited, especially with respect to a direct meta-analysis. It is likely that these same limitations are present in a comparative-analysis, and where features are present in both data sets a feature level comparison should also be performed in conjunction with an annotation level comparison.

## Concluding remarks

Analysis of both the data sets comparing denervation of skin with muscle and Crohn's with ulcerative colitis highlighted several biological processes shared across conditions as well as several that appear to be specific to each condition. These annotations provide a starting point for hypothesis generation and testing either by subsequent high-throughput experiments (or querying of analogous data sets) or directed experiments using potential targets derived from this analysis.

The hypothetical data analysis demonstrates that categoryCompare is able to illuminate processes highly relevant to the fundamental underlying biology that may be missed using a more traditional feature level analysis. This ability is dependent on the method of annotation enrichment employed (SBTF vs. RBTF), and the degree of noise. Although this analysis considered only data with identical classes of features, the annotation-level approach could enable meta-analyses of experiments with different types of features but shared annotations, such as genes and metabolites.

categoryCompare provides an easily extensible, general framework and interface for performing high-throughput data meta-analysis at the annotation level, in a commonly used programming environment with large amounts of available feature annotation data. Future work includes providing easy access to other types of feature annotations, calculating annotation enrichment for user provided annotations, and visual exploration of the feature—annotation relationships.

## Software availability

The current version of the categoryCompare package is available from http://bioconductor.org/packages/release/bioc/html/categoryCompare.html, while a development version is hosted on GitHub at https://github.com/rmflight/categoryCompare. It should be noted that the **ccPaper** branch of the version hosted on GitHub was used for all of this work. Many features from that branch will be available in the development version of the software package.

## Availability of supporting data

The data supporting the results reported are available in the ccPaper R package available from https://github.com/rmflight/ccPaper. The original raw data used (from denervated skin) is available from GEO as series GSE54356.

## Author contributions

Robert M. Flight conceived of the methodology, wrote, and maintains the Bioconductor package. Fahim Mohammad tested the package on various platforms, and edited the package vignette. Jeffrey C. Petruska, Lawrence D. F. Moon, and Benjamin J. Harrison posed the original question motivating the work, provided the skin denervation data set and biological interpretation. Eric C. Rouchka provided materiel, direction, and overall supervision of the project. Mary B. Bunge provided funding and consultation for the denervated skin microarray experimental design. All authors contributed to the writing of the manuscript.

## Funding

Funding was provided by the CDRF International Consortium on Spinal Cord Injury Research (Mary B. Bunge—author; Lorne M. Mendell and Fred H. Gage—acknowledged); (Both are now in acknowledgements rather than authors.) Kentucky Spinal Cord and Head Injury Research Trust (Grant 09-12A to Jeffrey C. Petruska); Paralyzed Veterans of America (Fellowship to Benjamin J. Harrison); National Institutes of Health (NIH) grants P20RR016481 (ECR), 3P20RR016481- 09S1 (Eric C. Rouchka, Robert M. Flight, Benjamin J. Harrison), P20GM103436 (Eric C. Rouchka), R21NS080091 (Jeffrey C. Petruska), and R21NS 071299 (Jeffrey C. Petruska); and Department of Energy contract DE-EM0000197 (Eric C. Rouchka). The article contents are solely the responsibility of the authors and do not represent the official views of the funding organizations.

### Conflict of interest statement

The authors declare that the research was conducted in the absence of any commercial or financial relationships that could be construed as a potential conflict of interest.
